# Verification of cuproptosis-related diagnostic model associated with immune infiltration in rheumatoid arthritis

**DOI:** 10.3389/fendo.2023.1204926

**Published:** 2023-07-20

**Authors:** Mingyang Jiang, Kaicheng Liu, Shenyi Lu, Yue Qiu, Xiaochong Zou, Ke Zhang, Chuanliang Chen, Yiji Jike, Mingjing Xie, Yongheng Dai, Zhandong Bo

**Affiliations:** ^1^ Department of Bone and Joint Surgery, Guangxi Medical University First Affiliated Hospital, Nanning, China; ^2^ Department of Rehabilitation, The Affiliated Hospital of Youjiang Medical University for Nationalities, Baise, China

**Keywords:** rheumatoid arthritis, cuproptosis, diagnostic model, immune infiltration, diagnostics

## Abstract

**Background:**

Rheumatoid arthritis (RA) is a chronic autoimmune disease closely related to inflammation. Cuproptosis is a newly discovered unique type of cell death, and it has been found that it may play an essential role in the occurrence and development of RA. Therefore, we intend to explore the potential association between cuproptosis-related genes (CRGs) and RA to provide a new biomarker for the treatment and prognosis of RA.

**Methods:**

Download GSE93777 datasets from the GEO database. Variance analysis was performed on the CRGs that had been reported. Then, the random forest (RF) model and nomogram of differentially expressed CRGs were constructed, and the ROC curve was used to evaluate the accuracy of the diagnostic model. Next, RA patients were subtyped by consensus clustering, and immune infiltration was analyzed in each subgroup to confirm the correlation between CRGs and abundance of immune cells. The expression levels of CRGs were verified by qRT-PCR.

**Results:**

Eight differentially expressed CRGs (DLST, DLD, PDHB, PDHA1, ATP7A, CDKN2A, LIAS, DLAT) were screened out by differential analysis to construct an RF model. The ROC curve proved that this model had good diagnostic accuracy. Based on the above eight significant CRGs, a nomogram was built to predict effective and high-precision results. The consensus clustering method identified two CRG patterns. Most of the immune cells were enriched in cluster A, indicating that cluster A may be related to the development of RA. Finally, qRT-PCR verified the expression of eight key genes, further confirming our findings.

**Conclusion:**

The diagnosis model of RA based on the above eight CRGs has excellent diagnostic potential. Based on these, patients can be divided into two different molecular subtypes; it is expected to develop a new treatment strategy for RA.

## Introduction

Rheumatoid arthritis (RA) is a chronic autoimmune disease closely related to inflammation. Recent statistics show that the global prevalence of RA is as high as 0.24% and an incidence of 20-45 per 100000 annually (1). In the current guidelines, the diagnosis of RA also depends on clinical manifestations, physical examination, and serological and imaging results. Unfortunately, although most RA patients have positive laboratory tests (rheumatoid serum factor and ACPA), about 1/3 of RA patients are negative (2). At the same time, no research has shown that a single pathological laboratory discovery or imaging method can diagnose RA, which brings significant challenges to diagnosing RA and affects the next step of treatment planning and prognosis rehabilitation. In addition, according to many studies, the treatment of RA still benefits from early diagnosis, early intervention, early treatment, and early referrals if necessary (3). There is no cure for RA; disease-modifying anti-rheumatic drugs (DMARDs) are the main recommended treatment for RA patients. However, long-term use of these drugs is bound to lead to adverse side effects, such as gastric ulcers, vomiting, heartburn, or gastrointestinal bleeding (4, 5). Therefore, reliable biomarkers are needed for early diagnosis, accurate prognosis, and therapeutic efficiency.

Cuproptosis is a unique programmed cell death in which intracellular copper accumulation leads to mitochondrial lipoprotein accumulation and Fe-S cluster protein instability (6). Reduction of copper ions (Cu^2+^) into Cu through ferredoxin 1 (FDX1) facilitates lipid acylation of mitochondrial proteins and excessive production of critical enzymes related to the TCA cycle, which in turn regulates key biological processes such as redox balance, iron metabolism, oxidative phosphorylation, and aberrant cell proliferation (7). Additionally, we discovered that patients with active RA had higher serum copper levels, which were negatively correlated with hemoglobin levels, an auxiliary disease indicator, and positively correlated with an erythrocyte sedimentation rate (ESR) and morning stiffness (8). A study reported that serum copper level could be used as an index of RA erosion activity (9). In previous studies, it has been pointed out that the relationship between cuproptosis and RA may be multifaceted, the inhibition of the neurite function of many kinds of immune cells leads to their excessive proliferation in RA, and some important neurite regulatory genes (such as PDHA1, PDHB, CDKN2A, and DLAT) be related to a variety of RA processes (10). But there are relatively few studies on the relationship between cuproptosis and RA at present, which indicates that cuproptosis is desired to become a new therapeutic target.

Therefore, our objective was to discover the potential role of cuproptosis in RA and offer theoretical references and direction for developing novel RA clinical treatments.

## Materials and methods

### Data sources

The GSE93777 (Transcriptome of whole blood and sorted immune cells from samples) datasets containing 133 normal and 315 RA samples were collected from the GEO database. We used Perl software to remove missing and duplicated data, followed by filtering and validating the entire dataset. The data was then annotated, and normalized. Using the R “limma” package to examine differentially expressed cuproptosis-related genes (CRGs) between normal and RA samples, |logFC| value > 1 and *P* < 0.05 were considered statistically significant. The “RCircos” package visualized the results, and the chromosome position of CRGs was depicted in the circle diagram. In addition, we analyzed the expression correlation between different CRGs.

### Construction of models

Lasso regression was used to further screen the CRGs in RA. Based on the chosen CRGs, we constructed random forest (RF) and support vector machine (SVM) models to predict RA patients’ incidence and obtain accurate results. Reverse cumulative distribution of residuals, boxplots of residuals, and ROC curves were used to assess the model’s accuracy. In our research, we finally selected the “RandomForest” package to establish an RF model and screen the key CRGs to predict the occurrence of RA. Then, the appropriate CRGs were screened with 10-fold cross-validation.

### Establishment of the nomogram

Using the “rms” package, construct a nomogram to forecast the incidence of RA based on the chosen key CRGs. After establishing the model, the decision curve analysis (DCA), calibration, and clinical impact curves were charted to assess the precision and effectiveness of the model.

### Molecular typing and PCA based on CRGs

We classified the eight screened CRGs using the “ConsensusClusterPlus” package’s consensus clustering method to investigate the relationship between CRG molecular subtypes and RA. PCA was used to assess the suitability of the classification.

### Infiltration of immune cells

We used ssGSEA to calculate the abundance of immune cells among the subgroups. Based on ssGSEA, the relationship between various CRGs and immune cell abundance was thoroughly examined using immune correlation analysis. CRGs significantly related to immune cell infiltration were selected for further study. The selected CRGs were divided into cohorts with high and low expression based on the median. The boxplots visualized the condition of immune cell infiltration between high and low-expression cohorts.

### Distinct CRG patterns and enrichment analysis for differentially expressed genes (DEGs) identification

The “limma” package in R was used to screen DEGs among CRG patterns according to the standard q value filter < 0.05. Then, we carried out the enrichment analysis of the differential genes by KEGG and GO analysis to explore the potential biological functions of DEGs in biological processes, molecular functions, and cellular components. The calculated *P*-value was corrected by Bonferroni after a threshold of corrected-p-value (q-value) ≤ 0.05. The results were visualized by the bubble diagram, and circle diagram.

### Establishment of the CRG signature

Considering individual differences, we used the PCA algorithm to evaluate the CRG sample’s score and characterize the CRG pattern. We then distinguished different CRG patterns by PCA.

### Quantitative reverse transcription polymerase chain reaction analysis

The cell lines used in the RA and control groups were CP-H248 and CP-H094 (human synovial fibroblasts [rheumatoid arthritis] and human synovial cells [normal control]), respectively. And they were obtained from cell bank (Procell. China). 10% fetal bovine serum was added to the DMEM mixture where the cells were grown. After the cell confluence rate reached 70% to 80%, all cells were cultivated in a 37°C, 5% CO_2_ saturated humidity incubator. After this, the cells were digested and subcultured with trypsin solution, and the cells in the logarithmic growth stage were taken for further qRT-PCR.

TRIzol kit regent (Invitrogen, USA) was used to extract the RNA from each group. The QuantiTect Reverse Transcription Kit (Qiagen, Germany) was used to reverse-transcribe the RNA into cDNA. Real-time fluorescence was used in quantitative PCR (qPCR) to gauge the amount of DNA present at each PCR cycle. SYBR-Green (Takara, Japan) was used to quantify real-time qPCR analyses. PCR amplification was performed in 40 cycles by denaturing at 95°C for 15s, annealing at 60°C for the 30s, and elongation at 60°C for 30s. Expression levels were normalized to GAPDH levels. Primer sequences are listed in **Table 1**. Data analysis adopted the 2^-ΔΔCT^ method.

**Table 1:** Forward and reverse sequences of primers.

Primer	Sequence (5′ to 3′)
ATP7A-F	5’TGCAGTCCCTCCATCTGGTA
ATP7A-R	AGACGCCTGGTTTTGGCTAC
LIAS-F	TGGTGTGACTACTTCAGAACCT
LIAS-R	GGAATAGGGCATGTGGATTTAGCA
DLD-F	CCGAACTGATGTAAGTAAACGGTC
DLD-R	GCCCACGTATTTGAGTTCCGTA
DLAT-F	GCGACGGGCTCAGAATGTAG
DLAT-R	CCCTGTAGTCACGCTGTTGC
PDHA1-F	GTCCGAGAGGCAACAAGGTT
PDHA1-R	GCTGTTCACCATCCTGTCCTT
PDHB-F	ATGCTCCTGCTGTTCGTGTC
PDHB-R	TTGCAGTACAAATCCAGGTGC
CDKN2A-F	CCACCCCGCTTTCGTAGTT
CDKN2A-R	AGTGAAAAGGCAGAAGCGGT
DLST-F	CGAATTTGTACTGGACCTGATAGGA
DLST-R	CAATCATGCGGTCTATCTGCC

### Statistical analysis

In this study, data processing and collation after the download was achieved *via* Perl software (Version 5.18.2). R version 3.6.1 undertook all data analysis. *P*-value < 0.05 was considered statistically significant if it wasn’t explicitly stated.

## Results

### The landscape of the 19 CRGs in RA

**Figure 1:**
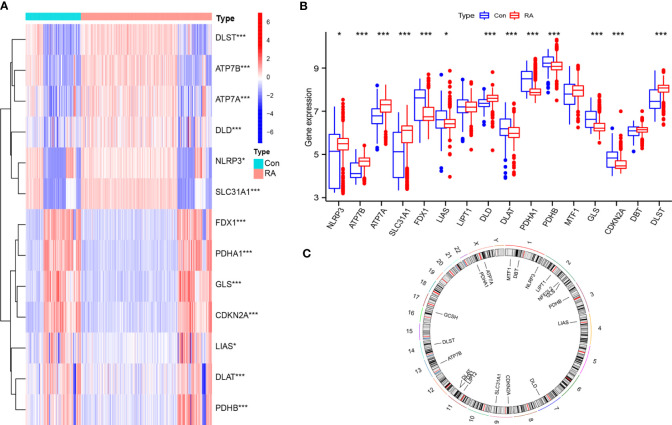
General situation of differential expression of CRGs between normal samples and RA samples. **(A)** 13 CRGs expression heat maps between the two groups; **(B)** 13 CRGs expression level in two groups; **(C)** The chromosomal locations of 19 CRGs. *P<0.05; **P<0.01; ***P<0.001.

Thirteen differentially expressed CRGs were screened **(** **Figures 1A, B** **)**. The expression of NLRP3, ATP7B, ATP7A, SLC31A1, DLD, and DLST was significantly up-regulated in RA samples, while the expression of FDX1, LIAS, DLAT, PDHA1, PDHB, GLS, and CDKN2A was down-regulated considerably in RA samples. **Figure 1C** illustrates the chromosomal positions of the 19 CRGs.

### Correlation between the different CRGs

**Figure 2:**
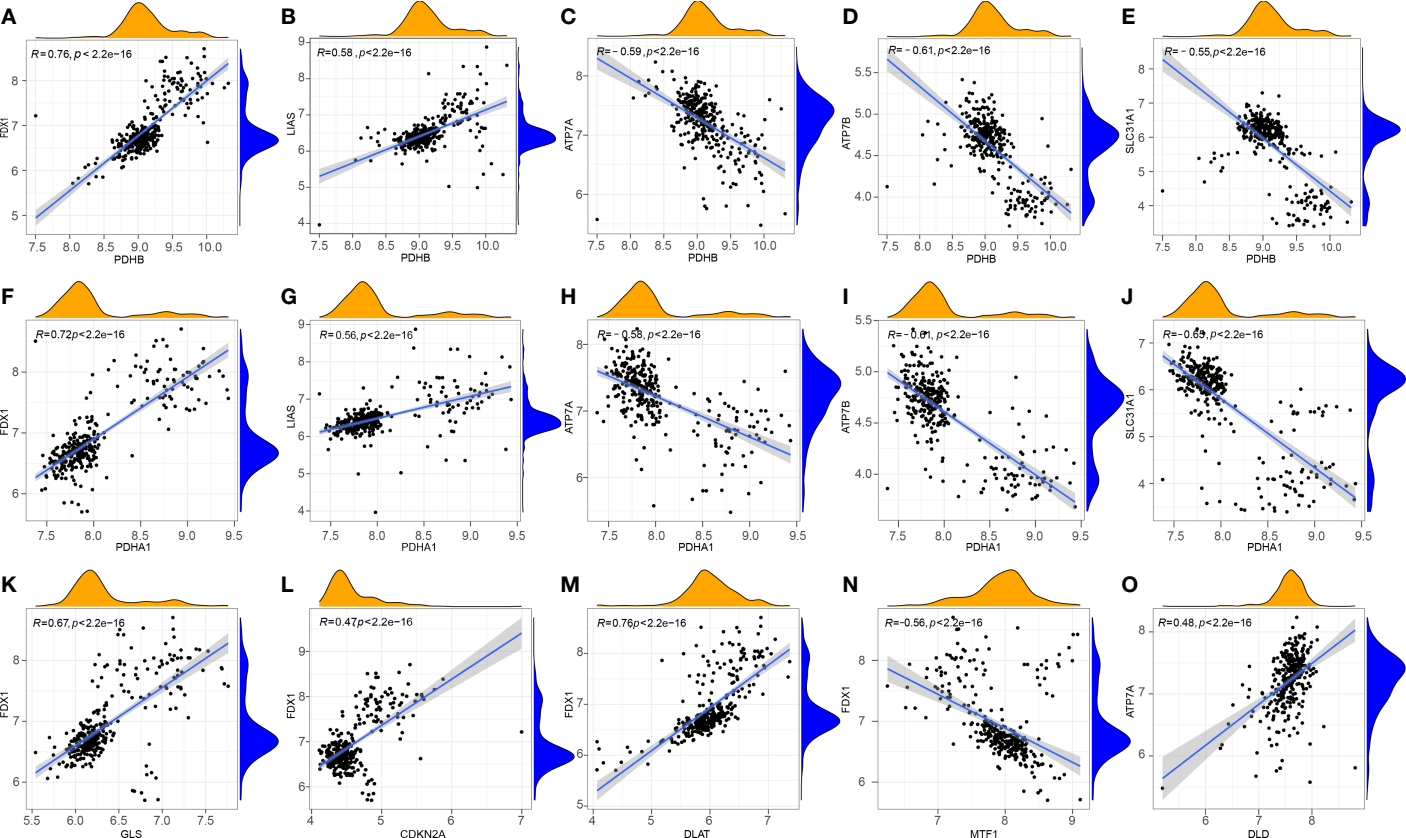
Correlation between CRGs in RA. **(A)** PDHB and FDX1; **(B)** PDHB and LIAS; **(C)** PDHB and ATP7A; **(D)** PDHB and ATP7B; **(E)** PDHB and SLC31A1; **(F)** PDHA1 and FDX1; **(G)** PDHA1 and LIAS; **(H)** PDHA1 and ATP7A; **(I)** PDHA1 and ATP7B; **(J)** PDHA1 and SLC31A1; **(K)** GLS and FDX1; **(L)** CDKN2A and FDX1; **(M)** FDX1 and DLAT; **(N)** FDX1 and MTF1; **(O)** DLD and ATP7A.

To explore the correlation between differentially expressed CRGs in patients with RA, the Pearson correlation analysis was performed using R statistical software **(** **Figures 2A–O** **)**. The results showed that DLD was positively correlated with ATP7A. PDHB was positively correlated with FDX1 and LIAS, and negatively correlated with ATP7B, ATP7A, and SLC31A1. PDHA1 was positively correlated with FDX1 and LIAS, and negatively correlated with ATP7B, ATP7A, and SLC31A1. FDX1 was positively correlated with GLS, CDKN2A, and DLAT, and negatively correlated with MTF1.

### Construction of the RF and SVM models

**Figure 3:**
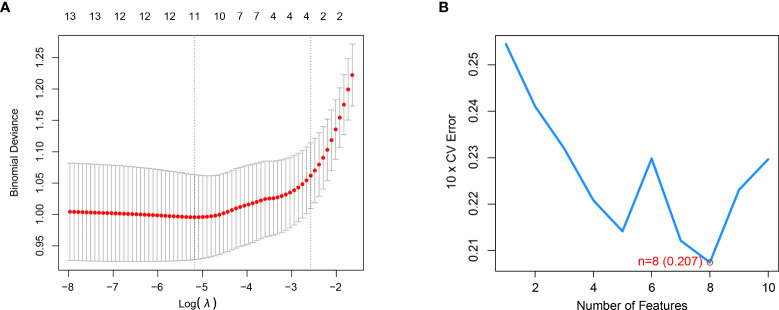
LASSO and SVM were applied to select significant CRGs. **(A)** LASSO showed that the three variables were left when the right dotted vertical line was drawn by one standard error of the minimum criteria. **(B)** SVM showed that the model of the top eight variables almost reached the lowest Root Mean Square Error value based on 10-fold cross-validation.

**Figure 4:**
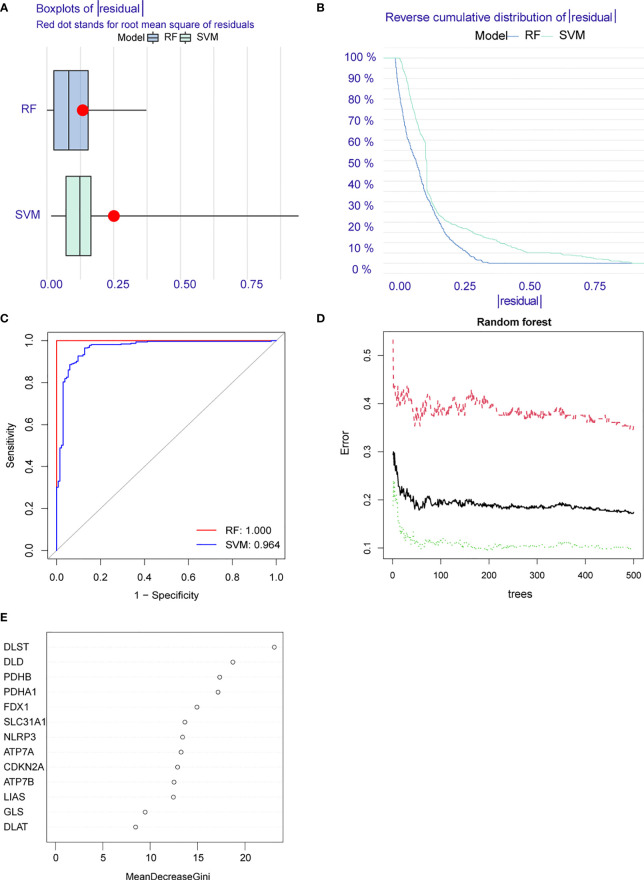
Construction of random forest (RF) and support vector machine (SVM) models. **(A)** Residual BoxPlot of RF and SVM, RF model showed a lower residual value; **(B)** Residual back-cumulative distribution diagram of RF and SVM, RF model showed a lower residual value; **(C)** ROC curve. The AUC of SVM is 0.964, The AUC of RF is 1.000; **(D)** A 10-fold cross-validation curve. Treat groups (red line), control groups (green line), and overall samples (black line). **(E)** The importance ranking of CRGs.

The Lasso regression analysis obtained a suitable number of variables when the Log λ=-2.67 **(** **Figure 3A** **)**. In the SVM, we selected eight CRGs (DLST, DLD, PDHB, PDHA1, ATP7A, CDKN2A, LIAS, DLAT) when the model reaches the lowest value of root mean square error (0.207) based on 10-fold cross-validation **(** **Figure 3B** **)**. Then we constructed the RF and SVM models to predict the disease based on the above eight differential genes. Comparing the residual boxplot **(** **Figure 4A** **)** and reverse cumulative distribution of residuals **(** **Figure 4B** **)** of the two models, the findings support the RF model’s superiority because of its minimal residuals. Meanwhile, the ROC curve was plotted to verify the precision of the two models further, and the area under the curve (AUC) also supports the above conclusion **(** **Figure 4C** **)**. The treatment groups, the control group, and the overall sample’s error levels were displayed on the 10-fold cross-validation curve **(** **Figure 4D** **)**. Meanwhile, we ranked the importance of above CRGs based on Gini index **(** **Figure 4E** **)**. The above results showed that the RF model based on screened CRGs could be used as the effective model to predict the occurrence of RA.

### Establishment of the nomogram

**Figure 5:**
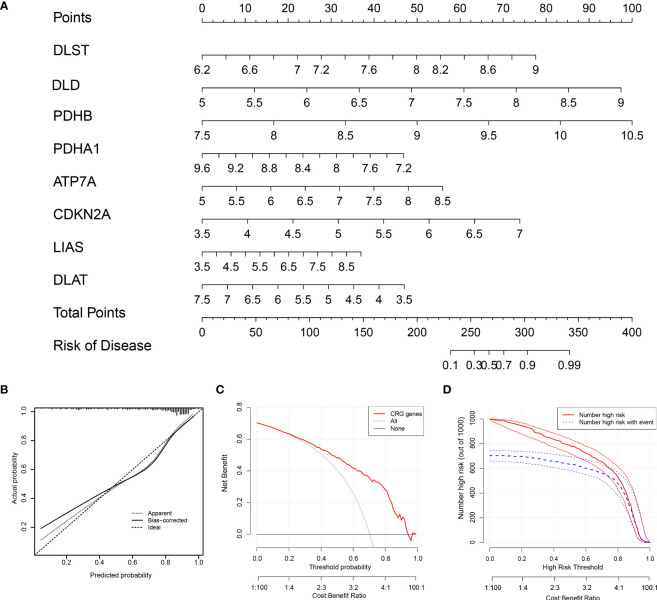
The nomogram construction. **(A)** The nomogram of the model; **(B)** The calibration curve proves the accuracy of the prediction ability of the new model. **(C)** The red, gray, and black lines related to cuproptosis in the Decision Curve Analysis (DCA) are biased. **(D)** The clinical impact curve proves that the model can effectively predict the results with high precision.

Using the “rms” package in R, we constructed a nomogram based on eight screened CRGs to predict the occurrence of RA **(** **Figure 5A** **)**. The calibration curve proved the accuracy of the model **(** **Figure 5B** **)**. The red line, gray line, and black line related to cuproptosis in the DCA are biased **(** **Figure 5C** **)**, which reveals the excellent decision-making ability of the nomogram. The clinical impact curve showed that the nomogram can effectively predict the results with high precision **(** **Figure 5D** **)**. This showed that our Nomogram had good prediction accuracy and reliability.

### Two CRG patterns identified by significant CRGs

**Figure 6:**
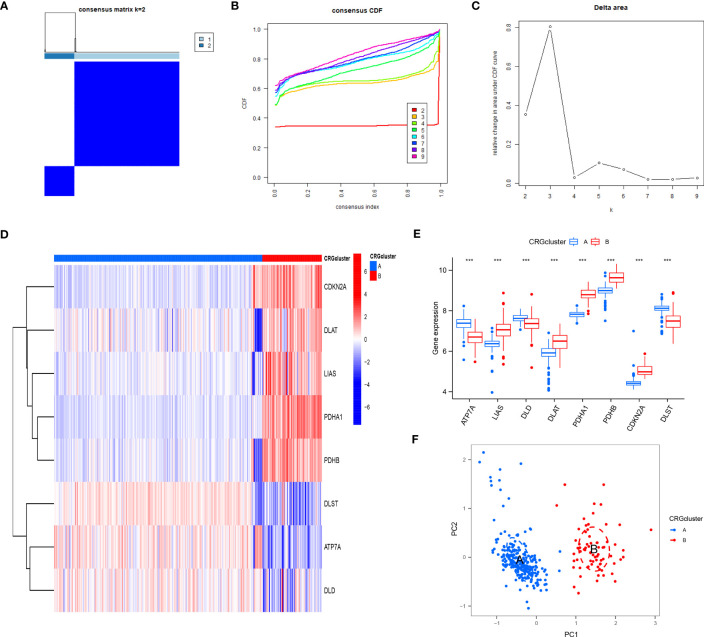
Consensus clustering of eight CRGs in RA patients. **(A)** Consensus matrix of eight CRGs with k = 2; **(B)** Cumulative distribution function (CDF) of consensus clustering; **(C)** Delta area plot of consensus clustering; **(D)** Differential expression heat map of eight CRGs in cluster A and cluster B; **(E)** Expression histograms of eight CRGs in cluster A and cluster B; **(F)** Principal component analysis (PCA) of eight CRGs showed that the above genes could be divided into two CRG patterns. *P<0.05; **P<0.01; ***P<0.001.

Based on eight candidate CRGs, using the “ConsensusClusterPlus” package, consensus clustering was applied to explore separate cuproptosis patterns, and two CRG patterns were identified **(** **Figures 6A–C**). The result indicated significant differences of eight CRGs in different subgroups. The expression levels of ATP7A, DLD, and DLST in cluster A were high, while LIAS, DLAT, PDHA1, PDHB, and CDKN2A were on the contrary **(** **Figures 6D, E** **)**. According to the results of PCA **(** **Figure 6F** **)**, eight CRGs could effectively distinguish into two CRG patterns.

**Figure 7:**
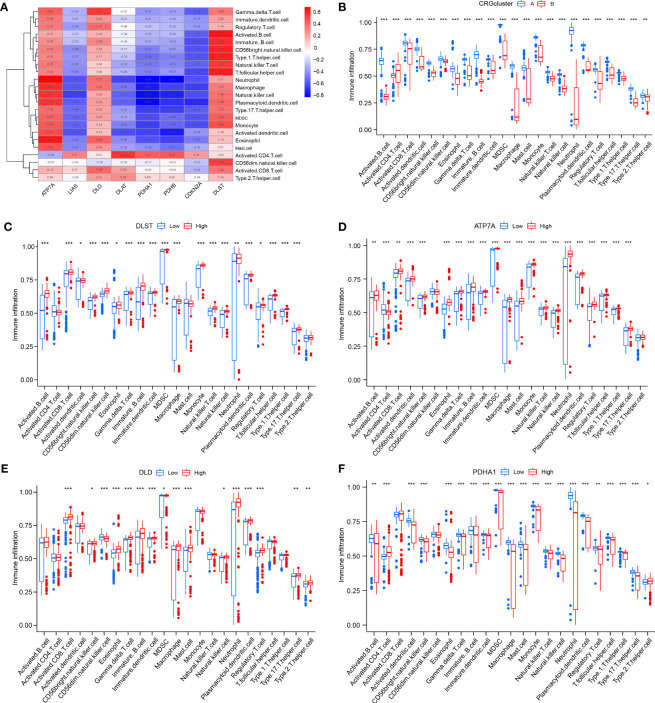
Analysis of immune cell infiltration. **(A)** The correlation between infiltrating immune cells and eight CRGs; **(B)** Differential immune cell infiltration between cluster A and cluster B; **(C)** Comparison of immune cell abundance between high and low DLST expression group; **(D)** Comparison of immune cell abundance between high and low ATP7A expression group; **(E)** Comparison of immune cell abundance between high and low DLD expression group; **(F)** Comparison of immune cell abundance between high and low PDHA1 expression group. *P<0.05; **P<0.01; ***P<0.001.

After that, the abundance of immune cells in the RA was analyzed by ssGSEA, and the correlation between eight candidate CRGs and immune cells was evaluated **(** **Figures 7A, B** **)**. The results showed a tight correlation between DLST, LIAS, DLD, PDHA1, ATP7A, and various immune cells. We divided DLST, LIAS, DLD, PDHA1, and ATP7A into high and low-expression groups and further analyzed the differential immune cell infiltration. Boxplots exhibited that compared with the low expression of DLST, DLD, and ATP7A, the immune cell infiltration of patients with high expression of DLST, DLD, and ATP7A increased; for the patients with low expression of LIAS and PDHA **(** **Figures 7C–F** **)**. The results were on the contrary, indicating that cluster A may be related to the occurrence and evolution of RA.

### Enrichment analysis and secondary clustering based on DEGs between diverse CRG patterns

**Figure 8:**
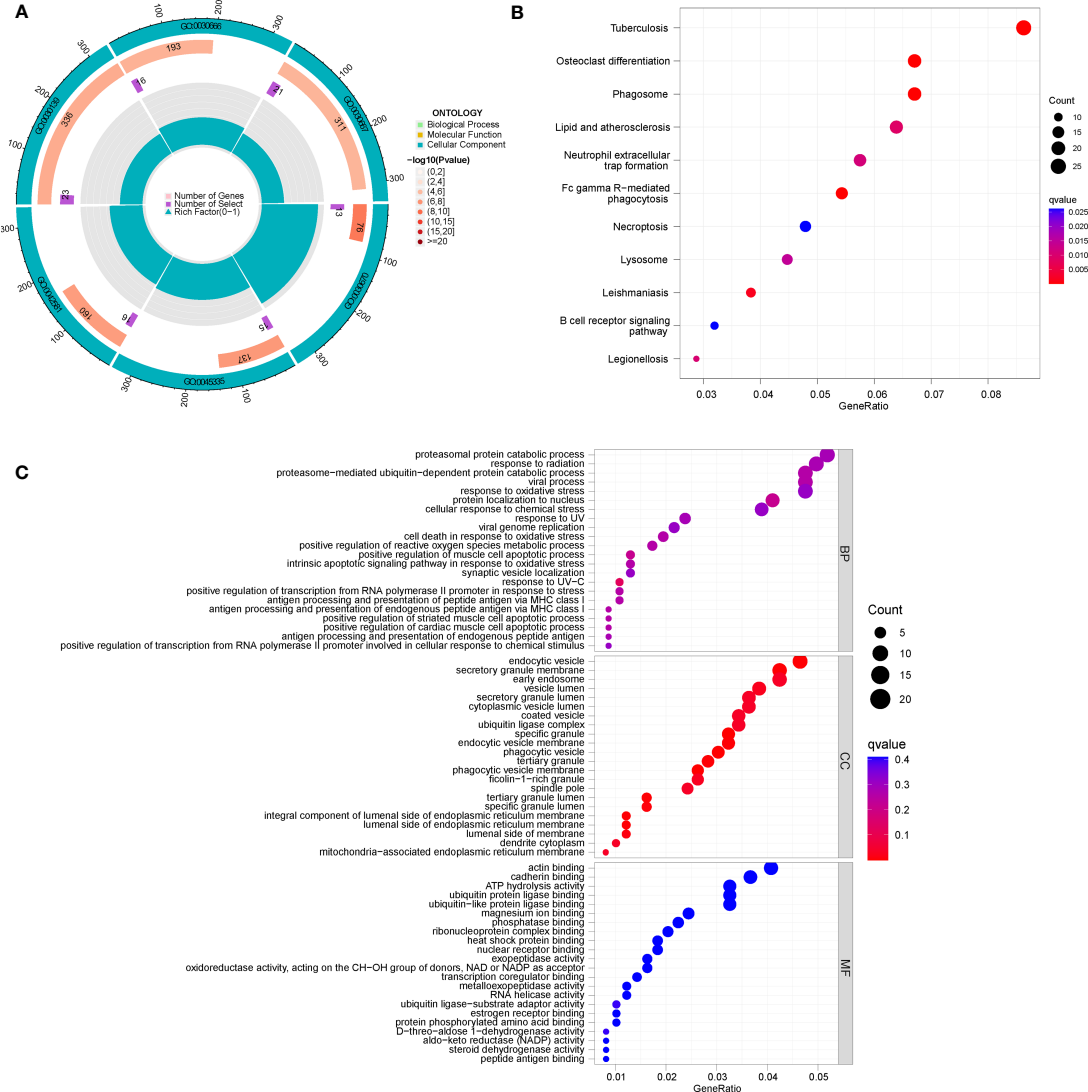
Enrichment analysis of DEGs between clusters (A, B), **(A)** GO circle diagram; **(B)** KEGG bubble chart; **(C)** GO bubble chart.

Under two distinct CRG patterns, 700 DEGs between clusters A and B were screened using the “limma” package. We found that the genes are mainly enriched in an endocytic vesicle (GO:0030139), secretory granule membrane (GO:0030667), endocytic vesicle membrane (GO:0030666), and specific granule (GO:0042581). GO and KEGG analysis was used to comprehend the potential mechanism of these DEGs in RA. It was observed that these DEGs were mainly enrich in proteasomal protein catabolic process, response to radiation, and viral process **(** **Figures 8A, C** **)**. It was found that the KEGG pathway was mainly enrich in Tuberculosis, Osteoclast differentiation, and Phagosome **(** **Figures 8B** **)**.

**Figure 9:**
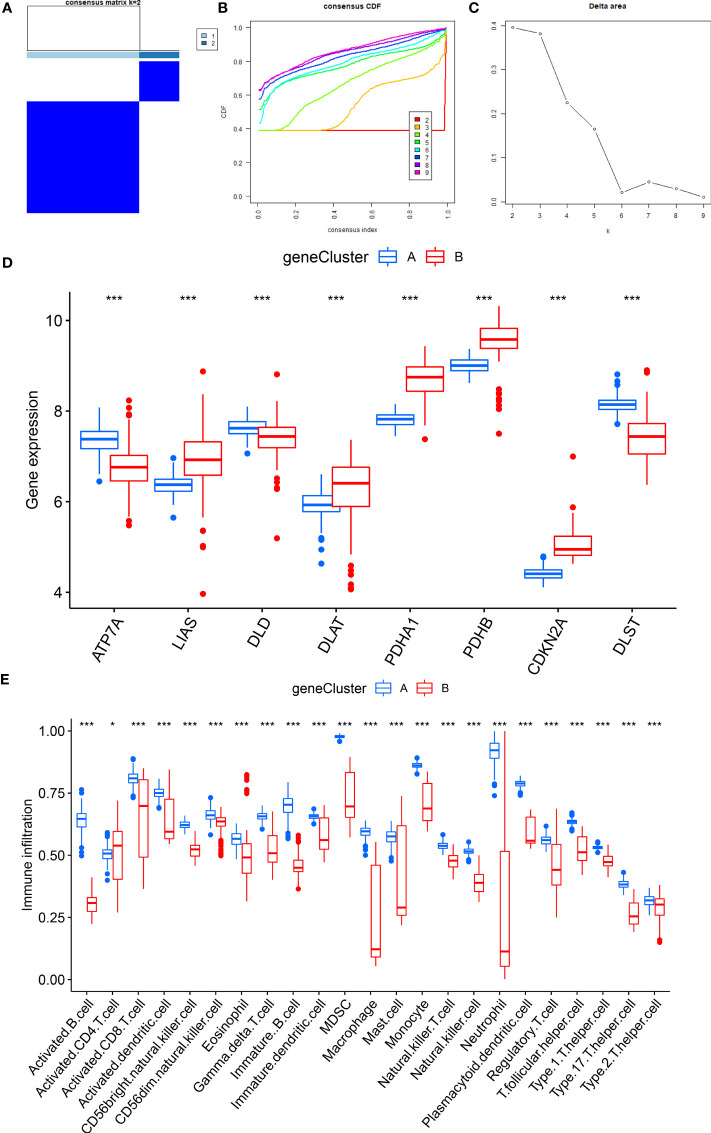
Consensus clustering of 700 cuproptosis-related DEGs. **(A)** Consensus matrix of 700 DEGs with k = 2; **(B)** CDF of consensus clustering; **(C)** Delta area plot of consensus clustering; **(D)** Expression of eight significant CRGs in cluster A and cluster B CRG gene models; **(E)** Different immune cell infiltration of DEGs in cluster A and cluster B CRG gene pattern. *P<0.05; **P<0.01; ***P<0.001.

**Figure 10:**
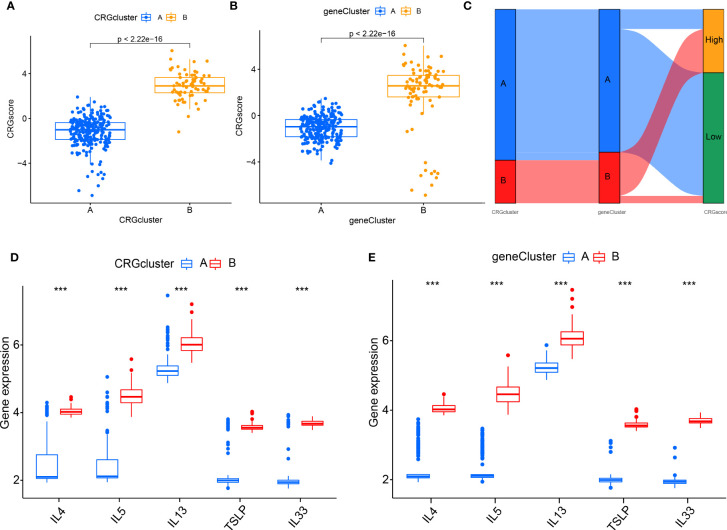
The role of different CRG patterns in diagnosing RA. **(A)** Differences in CRG scores between cluster A and cluster B; **(B)** Differences in CRG scores between gene cluster A and gene cluster B; **(C)** Sankey diagram showing the relationship between CRG patterns; **(D)** Differential expression levels of interleukin (IL)-4, IL-5, IL-13, IL-33 and thymic stromal lymphopoietin (TSLP) between cluster A and cluster B; **(E)** Differential expression levels of IL-4, IL-5, IL-13, IL-33, and TSLP between gene cluster (A) and gene cluster (B) *P<0.05; **P<0.01; ***P<0.001.

To further verify the CRG pattern, based on the above results of 700 DEGs, RA patients were divided into two different gene patterns by consensus clustering, which was found to be consistent with the grouping of CRG patterns **(** **Figures 9A–C** **)**. At the same time, the boxplot visualized the expression levels of eight significant CRGs and immune cell infiltration between gene clusters, which was similar to the expression results of our CRG patterns **(** **Figures 9D–E** **)**. It verified the accuracy of grouping. In addition, we quantified the CRG pattern using the PCA algorithm to calculate the sample’s CRG scores. We compared the CRG scores between two different CRG clusters or gene clusters. According to the findings, CRG cluster B or gene CRG cluster B had a higher risk score **(** **Figures 10A, B** **)**. The Sankey diagram **(** **Figure 10C** **)** illustrates the relationship between CRG patterns, CRG gene patterns, and CRG scores. These results were highly related to our above results and had strong consistency.

### The interaction between different patterns and cytokines

We examined the relationship between CRG patterns and CRG gene patterns, and RA to reveal further the correlation between CRG patterns and cytokines (**Figures 10D, E**). It was found that the results of CRG patterns and CRG gene patterns were consistent. The expression levels of IL-4, IL-5, IL-13, TSLP, and IL-33 in CRG cluster B or gene cluster B were higher.

### Validation of the expressions of key genes by qRT-PCR

**Figure 11:**
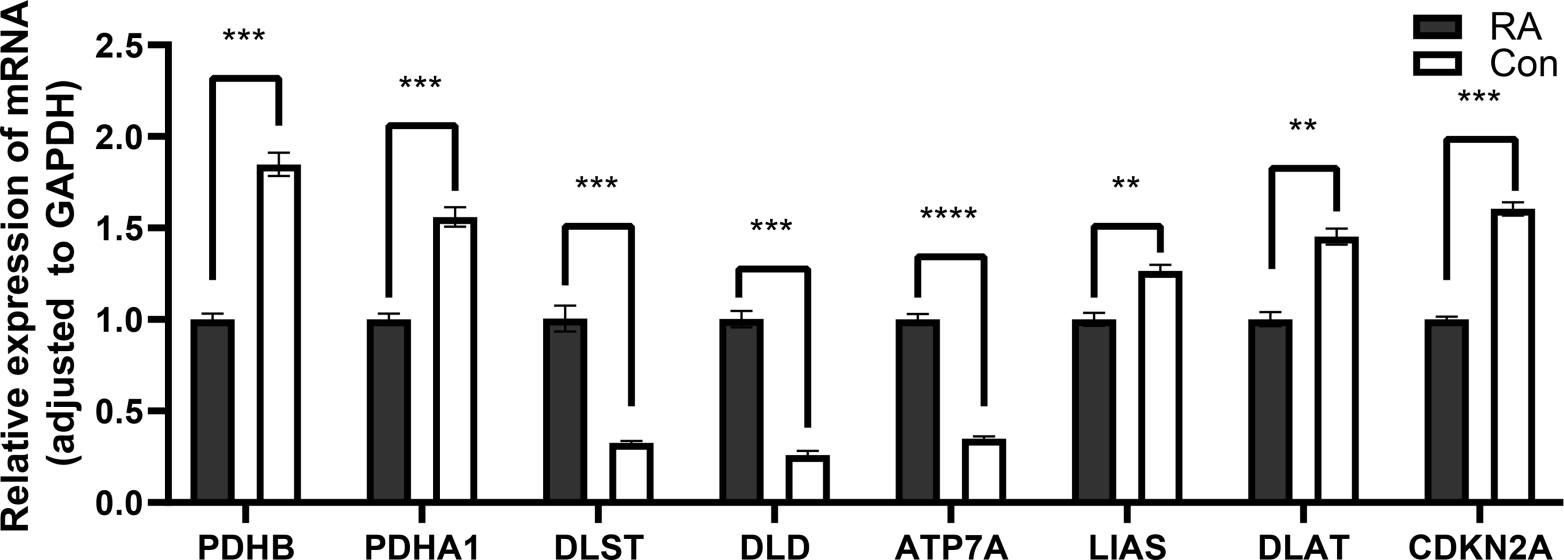
Detection of expression levels of eight key cuproptosis-related genes by qRT-PCR. *P<0.05; **P<0.01; ***P<0.001; ****P<0.0001.

Compared with control groups, the expression of DLST, DLD, and ATP7A in RA groups was significantly increased, while the expression of PDHB, PDHB1, LIAS, DLAT, and CDKN2A in RA groups was significantly decreased **(** **Figure 11** **)**.

## Discussion

It has been demonstrated that large concentrations of copper ions accumulated in cells can directly bind to lipid-acylated TCA cycle components, causing these proteins to aggregate and become dysregulated, blocking the TCA cycle, which further causes proteotoxic stress and results in cell death. Based on the 19 CRGs in previous studies, we screened eight CRGs (DLST, DLD, PDHB, PDHA1, ATP7A, CDKN2A, LIAS, and DLAT) in RA through Lasso regression and two machine-learning intersections to construct a nomogram for RA. In addition, we revealed 2 different cuproptosis modification patterns based on the expression of eight CRGs in RA. We also systematically examined the clinical importance of the different cuproptosis patterns and the immune cell infiltration level. Understanding the general biological characteristics of RA and the degree of immune cell infiltration under different patterns of cuproptosis regulation can help guide the selection of clinical immunotherapy regimens more effectively.

In the TCA cycle, cuproptosis binds copper to lipoylated enzymes, leading to toxic protein stress and cell death. Research has found that DLST is a TCA cyclic transferase that plays a role in the α-ketoglutarate(α-KG) dehydrogenase complex. It catalyzes the conversion of α-KG to benefit coenzymes to produce energy and synthesize macromolecules (11). At the same time, the TCA cycle plays a key role in cellular metabolism, which can meet the requirements of human biological energy, biosynthesis, and cellular redox balance (12). Studies have demonstrated that the risk of Primary Sjögren’s syndrome (pSS) is implicated with the overexpression of the DLD (13). In addition, we know that pSS is also an autoimmune disease dominated by chronic inflammation, and its pathogenesis is similar to that of RA. Therefore, we could speculate that the high expression of the DLD may be a new target for the therapy of RA. PDHA1 is an indispensable component of PDH and a rate-limiting enzyme complex that maintains the TCA cycle. Yetkin-Arik et al. have shown that tumor metabolism modulated by PDHA1 can affect cancer progression and metastasis (14). Studies have shown that PDHA1 plays distinct roles in different cancers. Elevated levels of PDHA1 were observed in uterine corpus endometrial carcinoma, cholangiocarcinoma, and lung carcinoma. In contrast, low expression of PDHA1 was observed in glioblastoma multiform, kidney renal papillary cell carcinoma, and thyroid carcinoma (15). The occurrence and development of all kinds of tumors are intimately correlated with human autoimmune infiltration and inflammation, so the expression of PDHA1 can be used as an index to forecast the incidence of diseases and assess the prognosis of various diseases. However, there are few reports on the relationship between these eight CRGs and RA in the existing literature. Therefore, we hope this paper’s results provide a theoretical basis for the follow-up research between CRGs and RA.

To further clarify the biological function of the two clusters. We carried out functional enrichment and typing of the differential genes between them. KEGG results showed they were significantly enriched in lipid and atherosclerosis, neutrophil extracellular traps (NETs), and necroptosis. Inflammation induced by excessive accumulation of lipids in arterial macrophages is a key factor in atherosclerosis (16). Inflammation promotes and enhances the development of atherosclerosis. The disorder of lipoprotein metabolism caused by low-density lipoprotein cholesterol (LDL) and apolipoproteins (Apo) B is also closely involved in the formation of atherosclerosis and eventually leads to related cardiovascular diseases. Studies have found that RA patients are at least twice as likely to suffer from cardiovascular disease as the general population. The inflammatory reaction related to RA can also accelerate the process of atherosclerosis, indicating that the pathophysiological mechanism of RA and atherosclerosis share a common pathway (17). NETs are a reticular structure composed of proteins released by neutrophils. In forming NETs, the imbalance between NETs formation and degradation may be related to autoimmune diseases (18). The structure of NET proteins is modified by histone, myeloperoxidase, and other antibacterial proteins. It has been found that many NET proteins are elevated in RA synovial fluid. These proteins contribute to cell infiltration and collagen degradation (19). Cuproptosis is a programmed cell death mediated. The interaction between RIPK1 and RIPK3 relates to the crosstalk of necrosis and apoptosis. It mediates not only the release of cell contents but also the occurrence of RA inflammation (20). This indicates that cuproptosis can induce inflammation related to RA, which in turn mediates the occurrence of RA and related complications. This is consistent with the above results, indicating that cuproptosis is highly related to the biological process of RA.

In this study, 315 patients with RA were divided into two CRG clusters. At the same time, the immune cell infiltration analysis also showed significant differences in almost of immune cells. Immune cell infiltration is more prominent in cluster A, suggesting they may have more significant benefits in treating RA. Our study found that T cells, dendritic cells (DC), macrophages, and neutrophils significantly differed between the two groups. RA is characterized by intra-articular immune cell infiltration. Previous studies have shown that innate immune cells (such as DC) and adaptive immune cells (such as T and B cells) mediate systemic autoimmune inflammatory response (21). It has been confirmed that the loss or functional deficiency of regulatory T (Treg) cells can lead to decreased self-tolerance in patients with autoimmune diseases, which is also one of the mechanisms leading to the progression of rheumatoid arthritis. As important immune cells, macrophages play an essential role in the initiation and regression of inflammation. The increase of macrophages is a prominent feature of inflammatory lesions. It has been reported that the increment in the number of macrophages in the synovium of patients with RA is an excellent sign that the disease is in the active stage. Joint erosion is correlated with the amount of synovial macrophage infiltration because they produce cytokines that increase inflammation and encourage the breakdown of cartilage and bone (22). Neutrophils are the first response cells to acute pathogenic injury. O’Neil L J et al. pointed out that neutrophils act on the joint synovium, regulate the innate and acquired immune responses of joints and the whole body, and play an essential role in the occurrence and development of RA (23). In addition, neutrophils can promote the production of modified autoantigens and further affect antigen-specific T cells and autoantibody responses. In summary, the level of immune cell infiltration may directly affect the effectiveness of immunotherapy in RA patients.

We further analyzed the immune cell infiltration among patients with high and low expression groups of DLST, DLD, ATP7A, and PDHA1. We found that B cells, activated CD8+ T cells, natural killer (NK) cells, MDSC, macrophage, and monocyte had significant significance in the positive correlation CRGs (DLST, ATP7A, DLD) in the high expression group, while in the negative correlation CRG (PDHA1), the expression level of these immune cells increased significantly in the low expression group. Some studies have shown that the accumulation of T and B cells is usually found in inflammatory synovial tissue of RA. NK, Treg, and helper cells are highly correlated with the activity of RA (24). In addition, B cells in the synovium of inflammatory RA are responsible for producing pathogenic antibodies such as ACPA so that they can be used as an essential target for treatment.

There are some limitations in this study. Firstly, the number of samples obtained from a single database is limited. In addition, the potential differences that cannot be ignored in different datasets affected the accuracy of our statistical analysis to some extent. Studies containing large samples are required to comprehensively assess the model constructed in this study and elucidate underlying mechanisms. Secondly, based merely on the mRNA levels, the assessment of cuproptosis cannot reflect the levels of proteomics and metabolomics. The posttranscriptional modifications and regulation of protein hydrolysis may impact protein activity. Thus, further studies based on multi-omics are needed to explore the mechanisms of cuproptosis deeply. Finally, more in-depth studies are required to go a step further to validate our results.

## Conclusion

In conclusion, based on the above eight CRGs, this study constructed a diagnostic model with strong prognostic prediction potential for diagnosing RA disease. We further clustered two CRG patterns, and RA patients with cluster A may have greater therapeutic benefits through immunotherapy. However, further research must be done to confirm the specific molecular mechanisms of CRGs in RA.

## Data availability statement

The original contributions presented in the study are included in the article/supplementary material. Further inquiries can be directed to the corresponding author.

## Author contribution

Conception and design: MJ, KL, SL. Administrative support: ZB. Provision of study materials or patients: ZB. Collection and assembly of data: YQ. Data analysis and interpretation: MJ, KL, SL. All authors contributed to the article and approved the submitted version.
